# Cell Cycle Regulatory Functions of the KSHV Oncoprotein LANA

**DOI:** 10.3389/fmicb.2016.00334

**Published:** 2016-03-30

**Authors:** Fang Wei, Jin Gan, Chong Wang, Caixia Zhu, Qiliang Cai

**Affiliations:** ^1^ShengYushou Center of Cell Biology and Immunology, School of Life Sciences and Biotechnology, Shanghai Jiao Tong UniversityShanghai, China; ^2^MOE & MOH Key Laboratory of Medical Molecular Virology, School of Basic Medicine, Shanghai Medical College, Fudan UniversityShanghai, China

**Keywords:** cell cycle, LANA, KSHV

## Abstract

Manipulation of cell cycle is a commonly employed strategy of viruses for achieving a favorable cellular environment during infection. Kaposi’s sarcoma-associated herpesvirus (KSHV), the primary etiological agent of several human malignancies including Kaposi’s sarcoma, and primary effusion lymphoma, encodes several oncoproteins that deregulate normal physiology of cell cycle machinery to persist with endothelial cells and B cells and subsequently establish a latent infection. During latency, only a small subset of viral proteins is expressed. Latency-associated nuclear antigen (LANA) is one of the latent antigens shown to be essential for transformation of endothelial cells *in vitro*. It has been well demonstrated that LANA is critical for the maintenance of latency, episome DNA replication, segregation and gene transcription. In this review, we summarize recent studies and address how LANA functions as an oncoprotein to steer host cell cycle-related events including proliferation and apoptosis by interacting with various cellular and viral factors, and highlight the potential therapeutic strategy of disrupting LANA-dependent signaling as targets in KSHV-associated cancers.

## Introduction

The eukaryotic cell cycle is divided into four phases: G1, S, G2, and M. The G1 phase is the first gap for cells to organize themselves prior to DNA replication. Any decisive events during G1 phase will determine whether the cell continues to proceed for division, pauses, or exits the cell cycle and enters the cell apoptosis pathway. The S phase is the stage for DNA synthesis, and hence genome duplication. The G2 phase is the second gap for cells to prepare the process of mitosis, and the associated cell division of two daughter cells, when the duplicated chromosomes are segregated into separated nuclei and cytokinesis. In addition, G2 phase also provides an opportunity for recognition and repair of damaged DNA. Thus, the G1 and G2 phase are called checkpoints for DNA replication and mitosis during cell cycle, respectively (Gabrielli et al., 2012). Strict regulation of cell division is critical for the normal development and maintenance of multicellular organisms. Loss of control of cell division will ultimately lead to cancer (Kastan and Bartek, 2004). In the past three decades, the studies of basic mechanism of cell cycle have led to a better understanding of how the molecular events required for cell division are controlled and coordinated (Gabrielli et al., 2012). The key elements in the basis of cell cycle regulation are the periodic synthesis and destruction of cyclins, which associate with or activate cyclin-dependent kinases (Cdks) (Dai and Grant, 2011). Although at least 16 cyclins and 9 Cdks have been identified in mammalian cells, not all cyclins and Cdks is necessary to regulate the cell cycle, some have been shown act as regulators of transcription, DNA repair or apoptosis (Johnson and Walker, 1999). In addition to the interaction between cyclin and Cdks, there are several levels of regulation including cyclin-dependent kinase inhibitors (CdkIs) and ubiquitin-mediated proteolysis which are also involved in controlling the activity of Cdks during the cell cycle (Kastan and Bartek, 2004).

Manipulation of the host cell cycle is a frequent strategy for viruses to evade host cells, presumably in order to achieve a cellular environment favorable for their replication (Nascimento et al., 2012). Due to the complex and interactive nature of intracellular signaling pathways in controlling cell division, which could provide many opportunities for viral manipulation, the important effect of viral regulation on cell cycle dynamics are the consequences for driving neoplastic transformation. This offers a rational approach to the control of virus causing cancers (Gabrielli et al., 2012). The study of host evasion strategies for cell cycle manipulation evolved by viruses will undoubtedly reveal new control mechanisms and their corresponding cellular signaling pathways.

**FIGURE 1:**
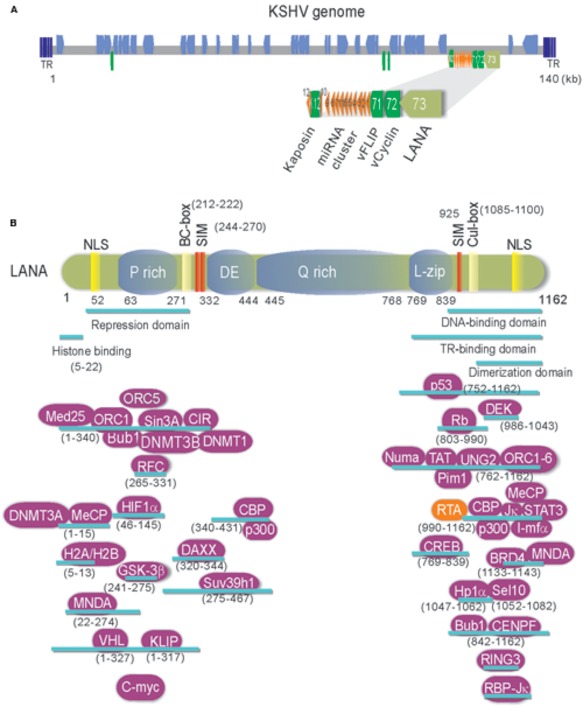
**(A)** Schematic and location of the KSHV latent genes including miRNA cluster. Bottom: The major latency locus (ORF73/LANA, ORF72/v-Cyclin, ORF71/vFLIP, and K12/Kaposin) of KSHV is shown in an enlarged view. Position of 12 pre-miRNA cluster is shown in red triangle. **(B)** The structure and functional motifs of Latency-associated nuclear antigen (LANA). LANA consists of 1162 amino acids. Numbers indicate the amino acids (aa). Repetitive regions and key motif of LANA are noted. P, Proline; DE, Aspartic acid and Glutamic acid; Q, Glutamine; L, Leucine; NLS, nuclear localized sequence; BC, Elongin B and C; Cul: Cullin5; SIM, SUMO-interacting motif. The binding regions of LANA-associated cellular and viral proteins are listed at the bottom panel.

Kaposi’s sarcoma-associated herpesvirus (KSHV), also known as human herpesvirus type 8 (HHV-8), is a gamma-herpesvirus associated with several human malignancies including Kaposi’s sarcoma (KS), primary effusion lymphomas (PEL), and multicentric Castleman’s disease (MCD) (Dupin et al., 1999). As shown in **Figure 1A**, the KSHV genome is an approximately 140 kb long unique coding region (LUR) that is flanked by multiple, non-coding terminal repeat (TR) units with high GC content (Russo et al., 1996; Ohsaki and Ueda, 2012). The LUR encodes about 90 open reading frames (ORFs), 12 microRNAs and several ncRNAs (Russo et al., 1996; Toth et al., 2013). Like all herpesviruses, KSHV exhibits two distinct phases of infection: latency and lytic replication. In primary infection, KSHV enters a latency whereby the viral genome circularizes and exists as nuclear episome through multiple host cell divisions. During latent infection, only a subset of viral genes including latency-associated nuclear antigen (LANA, ORF73), v-Cyclin (ORF72), v-FLIP (ORF71), and Kaposin (K12) are expressed (**Figure 1A**). Upon stimulation such as chemical agents or environmental stress, KSHV could be reactivated from latency to lytic replication and in turn produce infectious virion progeny (Yu et al., 1999; Davis et al., 2001).

## LANA Encoded by KSHV is a Multi-Functional Oncoprotein

Many evidences have shown that KSHV establishes a stable latent infection which plays an essential role in KSHV-induced malignancies and pathogenesis (Dupin et al., 1999; Katano et al., 2000; Parravicini et al., 2000). Serological analysis of the infected cells by immunofluorescence and immunohistochemistry indicated that LANA is one of only a few latent proteins consistently present in all KSHV-infected tumor cells of KS, PEL, and MCD (Dupin et al., 1999; Katano et al., 2000; Parravicini et al., 2000). It has been well demonstrated that LANA is a multi-functional protein with approximately 1162 amino acids in length, through directly or indirectly interacting with many other molecules in different signaling pathways including apoptosis, cell proliferation and gene transcription (**Figure 1B**). Many studies have shown that LANA contains unique nuclear localization signal (NLS) and is localized in the nuclei and interacts with host cellular DNA and KSHV genome in a punctate pattern during interphase and mitosis (Ballestas et al., 1999; Cotter and Robertson, 1999). This indicates that LANA is a cell-cycle related protein. LANA contains a large internal region of acidic and glutamine-rich repeat, and separating the amino and carboxyl terminal region of LANA, although it has been noted that the length of the internal repeat region varies between different KSHV isolates (Gao et al., 1999). LANA encodes various functional motifs to specifically recruit different target molecules. For examples, LANA has a chromosome-binding motif within 5–13 amino acids to bind with histones H2A/H2B (Barbera et al., 2006), a motif to recognize DNA sequence within the TR region of KSHV (Srinivasan et al., 2004; Kelley-Clarke et al., 2009), a region for LANA oligomerization (Komatsu et al., 2004), a SOCS-like box motif to recruit EC_5_S (Elongin BC-Cullin5) ubiquitin complex (Cai Q.-L. et al., 2006), a SIM motif to bind with SUMO molecules (Cai et al., 2013), and so on. Interestingly, the phenomenon that LANA drives transgenic mice developing splenic follicular hyperplasia (Fakhari et al., 2006), and transforms primary REF cells when conjunction with h-Ras (Radkov et al., 2000), as well as LANA upregulates the transcriptional activity of human telomerase promoter through interaction with Sp1 (Knight et al., 2001; Verma et al., 2004), supporting the notion that LANA acts as an oncoprotein to contribute to the pathogenesis of KSHV infection. In regard to how LANA plays a critical role in KSHV episome persistence, DNA replication and gene transcription, as well as program switch of latency and lytic replication, two reviews have been summarized recently (Ballestas and Kaye, 2011; Uppal et al., 2014). However, although it is clear that LANA binds to nucleosomal proteins throughout the cell cycle, how LANA associates with the regulators of cell cycle to driving cell proliferation remains elusive. Here, we summarize and highlight the recent progression of cell cycle regulatory functions of LANA.

## LANA Deregulates Cellular Oncoproteins and Growth Suppressors

**FIGURE 2:**
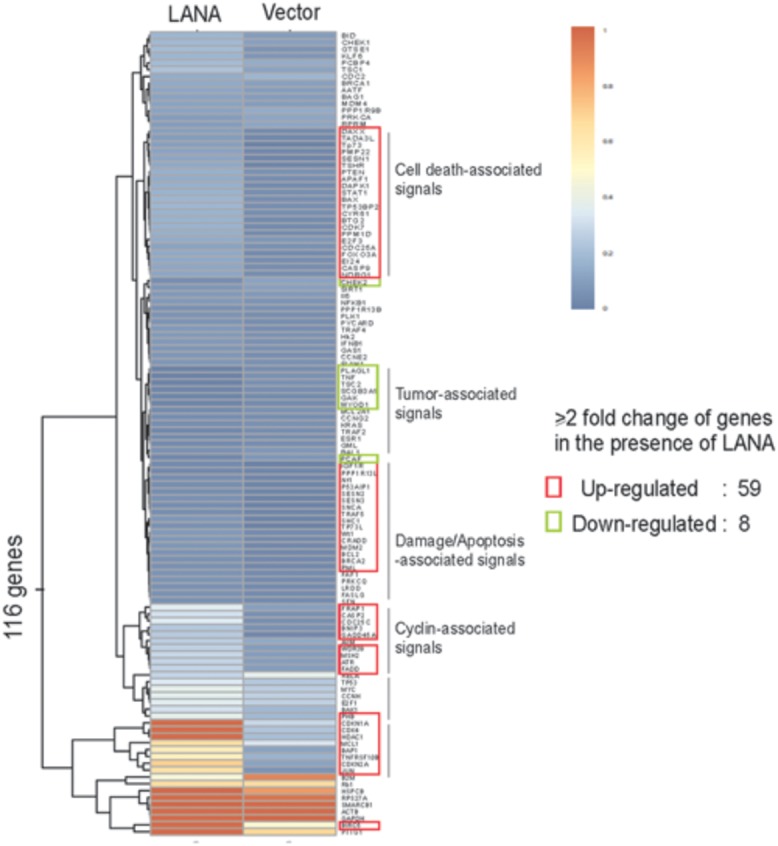
**Hierarchical clustering of genes profiling related to cell cycle in BJAB cells with LANA-RFP stable expression or RFP vector alone.** Data analysis revealed that 67 out of 116 genes are changed by over twofold in their expression in the presence of LANA.

Both control of cell cycle checkpoints and inhibition of apoptosis are hallmarks of tumor cell proliferation including KSHV-infected KS and PEL cells. Many studies have shown that LANA not only blocks tumor suppressor pathways, but also enhances expression of oncogenes which involve in cell cycle regulation, supporting the role of LANA in cell transformation and growth. For examples, the inhibitors of DNA binding (Id) have been demonstrated to involve in cell cycle regulation through deregulating expression of p21 (Nickoloff et al., 2000). Moreover, Id-1 could directly interact with the p16 promoter to drive cell proliferation (Alani et al., 2001). The introduction of LANA in endothelial cells dramatically increased Id-1 expression, suggesting that LANA may drive cell proliferation by targeting Id-1 expression (Tang et al., 2003). Many other oncoproteins associated with prolonged cellular survival are also affected by LANA. For examples, LANA activates telomerase expression (Verma et al., 2004), stabilizes c-myc and HIF1α oncoproteins and promotes its transcriptional activity (Cai Q. et al., 2006; Bubman et al., 2007; Cai et al., 2007; Liu et al., 2007b), increases level of ICN in Notch signaling pathway (Lan et al., 2007), upregulates expression of Aurora Kinase (Cai et al., 2012), as well as associates with and upregulates Pim1 kinase activity (Bajaj et al., 2006; Cheng et al., 2009). On the other hand, LANA also functions as a component of E3 ubiquitin ligase to target tumor suppressors like p53 and VHL for degradation, which creates a favorable environment for cell growth. LANA also affects the tumor suppressor p73 stability and subnuclear localization to contribute to the survival of PEL cells (Santag et al., 2013). Due to activation of TGF-β signaling pathway in many cell types results in inhibition of cell growth and induction of apoptosis, LANA suppresses the promoter of TGF-β type II receptor (TβRII) through epigenetic silencing (Di Bartolo et al., 2008), which may lead to cell proliferation by activation of c-myc, p15, and Cdc25A. To further explore the role of LANA on cell cycle regulation, our recent studies revealed that LANA upregulates transcription of 59 of 116 cell-cycle genes (including CDK4, Cdc25A/C, BAX, and BCL2), and only downregulates eight genes (**Figure 2**) (Cai et al., 2010; Gan et al., 2015). This indicates that LANA plays a positive instead of negative regulation on process of cell progression.

## LANA Manipulates G1-S Progression

**FIGURE 3:**
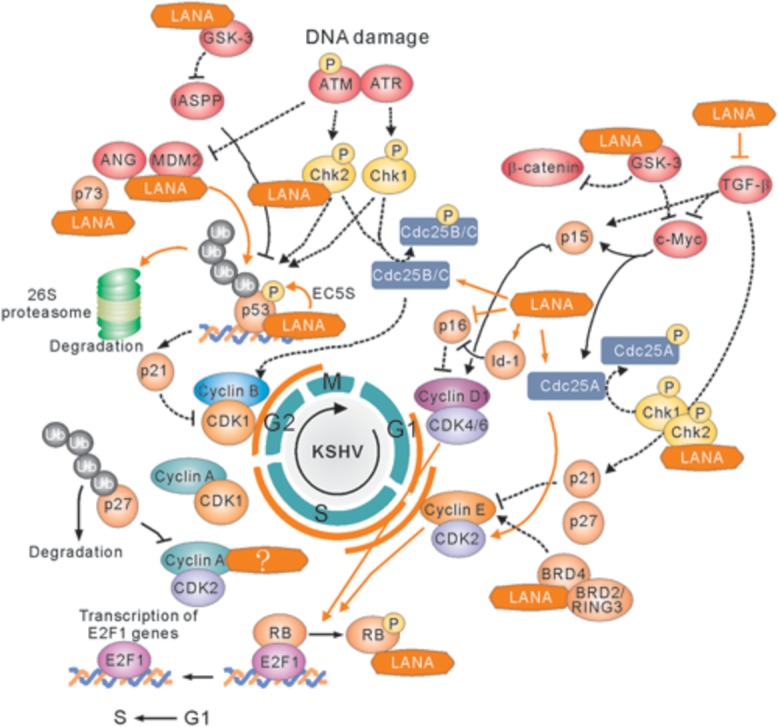
**Latency-associated nuclear antigen-mediated deregulation of cell cycle.** LANA associates with many cellular proteins including GSK-3, c-Myc, Chk2, BRD4, and BRD2/RING3, to activate Cyclin D1-CDK4/6 and Cyclin E-CDK2 complex, which results in the hyperphosphorylation form of retinoblastoma protein (RB). Hypersphophorylation of RB prevents its interaction with E2F, and releases E2F to activate expression of genes required for entry into the S phase. LANA also interacts with RB to facilitate G1/S phase transition. On the other hand, LANA not only recruits E3-ligase complex (including Elongin BC-Cullin 5, EC_5_S) or MDM2 to target p53 for ubiquitin-mediated degradation, but also associates with many p53-associated pathway regulators including p73, iASPP, and Chk2 to induce Cyclin B-CDK1 complex for driving G2/M phase transition. The positive and negative regulation by LANA is shown by solid orange line. The cellular signaling pathway blocked by LANA is indicated by dot line.

Upon growth factors such as epidermal growth factor (EGF) and insulin-like growth factor (IGF), the sequential activation of the two kinase complexes, Cdk4/6-cyclin D, and Cdk2-cyclin E, is the key event that leads to cell cycle progression (**Figure 3**). These activated complexes could phosphorylate tumor suppressor retinoblastoma (RB), and in turn dissociate E2F from RB and accumulate E2F. In the LANA-expressing cells, LANA interacts with RB and enhances the transcriptional activation of E2F-responsive genes (Hume and Kalejta, 2009). In addition, LANA has also been shown to interact with glycogen synthase kinase (GSK-3β), a kinase involved in phosphorylation and subsequent degradation of many cell-cycle regulators including c-Myc and Cyclin D (Fujimuro et al., 2003; Bubman et al., 2007; Liu et al., 2007a,b). Recent studies showed that the interaction of GSK-3 with LANA could lead to the accumulated expression of iASPP and in turn degradation of p53 for cell growth (Woodard et al., 2015). Our previous studies further demonstrated that p53 can be degraded by LANA-mediated recruitment of the cellular EC_5_S (Elongin BC-Cullin 5-Rbx1) ubiquitin complex (Cai Q.-L. et al., 2006), as well as involvement of the Serine/Threonine oncogenic kinase Aurora A (Cai et al., 2012).

In response to DNA damage, entry of cells into S phase is prevented by activation of the two transducing kinases, ATM/ATR and Chk1/Chk2, and followed by Cdc25A phosphorylation and p53 activation. Phosphorylated Cdc25A pathway has a faster inhibitory impact on the cell cycle progression (Lukas et al., 2004). In contrast, both Chk1/Chk2 and ATM/ATR-mediated phosphorylation on Serine 15 of p53 signaling can further prolong G1 arrest. Recent studies showed that LANA directly interacts with Chk2 to block ATM/ATR-mediated apoptosis and potential activation of Cdc25A (Kumar et al., 2014). Meanwhile, Mdm2 (the negative regulator of p53) is inactivated by ATM/ATR, and association of p53 with p300 results in increased transcriptional activity of p300. The p53-mediated induction of downstream genes including p21 blocks the G1/S progression which promoted by Cdk2-cyclin E kinase expression. The inhibitors of p53-MdM2 interactions including nutlin-3 interfere with the formation of Mdm2-p53-LANA complex and cause G1 arrest and apoptosis of PEL cells (Petre et al., 2007; Sarek et al., 2007; Ye et al., 2012), further supporting the notion that modulation of p53-dependent pathways by LANA is critical for KSHV to prevent cell cycle arrest and apoptosis.

In addition, LANA has been shown to bind and block p53-mediated transcriptional activity and in turn inhibit p53-induced cell apoptosis (Friborg et al., 1999; Si and Robertson, 2006; Lu et al., 2009). It has been demonstrated that LANA can also positively regulate cell cycle-dependent promoters, and promote the G1/S transition of cells by overcoming the serum starvation, overexpression of cyclin-dependent kinase inhibitor p16, or BRD4 and BRD2/RING3-induced G1 cell cycle arrest (Platt et al., 1999; Direkze and Laman, 2004; An et al., 2005; Viejo-Borbolla et al., 2005; Ottinger et al., 2006). Moreover, Disruption of Annexin A2 with LANA results in downregulation of cell cycle-asociated CDK6, cyclin D, E, and A protein, indicating that LANA upregulates cyclin D, E, and A proteins during the G1/S phase of cell cycle (Paudel et al., 2012a). In consistent, to maintain the viral genome in each cell cycle, LANA recruits proliferating cell nuclear antigen (PCNA) via Bub1 and replication factor C (RFC) to initiate DNA replication of viral genome during S phase, and enhances survival of KSHV-infected cells in response to UV-induced DNA damage (Sun et al., 2014, 2015). In addition, to enhance the survival of KSHV-infected cells, LANA upregulates the angiogenic multifunctional protein angiongenin (ANG) (Sadagopan et al., 2009). Further studies showed that LANA interacts with ANG/annexin A2 to contribute to the binding of LANA with p53 (Paudel et al., 2012a,b).

## LANA Efficiently Disrupts the Block to the G2/M Checkpoint

DNA damage is a common phenomenon through exposure to a variety of environmental stresses, including abnormally low oxygen, or nutrients. Before cells enter mitosis, the G2/M checkpoint responds directly to DNA damage by repairing DNA breaks or alternately by holding cell cycle progression and/or by undergoing programmed cell death. The key target of G2 arrest is the mitosis promoting complex Cdk1-cyclin B. In response to different type of DNA damage, the ATM-Chk2 or the ATR-Chk1 signal pathway is activated to arrest the cell in G2 phase by blocking activation of Cdk1-Cyclin B or inhibition of Cdc25B/C phosphatase (an enzyme normally activates Cdk1 at G2/M transition). The increased phosphorylation of Cdc25B/C is observed during G2 arrest. Both Chk1 and Chk2 kinase are able to phosphorylate Cdc25B/C in response to DNA damage. The ATM/ATR kinase not only activates Chk1 and Chk2 but also phosphorylates p53 on its serine 15. Phosphorylation of p53 prevents p53 binding with MdM2, thereby stabilizing p53. The stabilized p53 in turn up-regulates the Cdk inhibitor p21 at the G1 checkpoint, which eventually leads to cell arrest or apoptosis. Although no literatures reported that LANA could directly target Cdc25B/C or Cdk1-cyclin B complex, many molecules [including p73 (Santag et al., 2013), ANG (Sadagopan et al., 2009), Mdm2 (Sarek et al., 2007), and GSK-3 (Woodard et al., 2015)] have been shown to interaction with LANA for blocking p53-mediated apoptosis during G2/M checkpoint. Recent studies showed that LANA upregulates the expression of survivin, and recruits Aurora kinase B to induce phosphorylation of Survivin at Tyrosine 34 (Lu et al., 2009, 2014), which potentially drive host cell proliferation during mitosis. Moreover, in human B cells, it has been demonstrated that LANA directly interacts with Chk2 (the ATM/ATR signaling effector) to relieve the nocodazole-induced G2/M checkpoint arrest (Kumar et al., 2014). This indicates that LANA also involve in the deregulation of G2/M checkpoint during cell progression (**Figure 3**).

## Future Perspective

In the view of the facts that in addition to promoting cell cycle arrest, most members of herpesviruses family also activate several factors to induce cell cycle progression. It is of importance and complex to elucidate how KSHV-encoded LANA balances the interaction between herpesvirus and cell cycle regulatory mechanisms. In the past two decades, numerous studies have shown that LANA is a multifunctional oncoprotein ubiquitously expressed in the KSHV-infected cells, and modulates various cellular pathways to drive cell proliferation. To seek the chemical compounds to efficiently block the KSHV-driven cell proliferation and its associated cancers, Gao and Schulz groups have shown that the small-molecule inhibitors Nutlin-3 and RETRA, which disrupt the interaction of p53 and p73 with MDM2, are efficient to individually induce apoptotic cell death in a p53 and p73-dependent manner (Ye et al., 2012; Santag et al., 2013). Glycyrrhizic acid (GA), a triterpenoid compound shown to inhibit the lytic replication of herpesviruses, is able to reduce the expression of LANA and leads to G1 cell cycle arrest and p53-mediated apoptosis of PEL cells (Curreli et al., 2005). Gamma-secretase inhibitor targeting LANA-mediated Notch pathway also inhibits cell growth and death (Liu et al., 2010). Taken together, these studies now clearly show that in addition to regulating transcription, chromatin remodeling, episome maintenance, DNA replication, and control of latency and lytic reactivation, LANA also plays a critical role in KSHV-mediated tumorigenesis by regulating cell cycle machinery. Understanding the role of LANA in regulation of cell proliferation, particularly on regulation of episome replication and segregation during cell cycle, will lead to a better understanding of cellular growth control processes, which will open an opportunity target to prevent and treat KSHV-associated malignancies.

## Author Contributions

FW, QC wrote, and reviewed the manuscript, JG, CW, CZ analyzed data.

## Conflict of Interest Statement

The authors declare that the research was conducted in the absence of any commercial or financial relationships that could be construed as a potential conflict of interest.
